# RHBDF1 promotes PERK expression through the JNK/FoxO3 pathway in breast cancer cells: RHBDF1 promotes PERK expression through the JNK/FoxO3 axis

**DOI:** 10.3724/abbs.2024163

**Published:** 2024-10-17

**Authors:** SungJu Ryu, Hui Long, Xiaojing Quan, UnChol Kim, Wenwen Zhao, Yuanyuan Song, Luyuan Li, Zhisong Zhang

**Affiliations:** 1 State Key Laboratory of Medicinal Chemical Biology and College of Pharmacy Tianjin Key Laboratory of Molecular Drug Research Nankai University and the Haihe Laboratory of Cell Ecosystem Tianjin 300350 China; 2 Institute of Microbiology State Academy of Sciences Pyongyang Democratic People’s Republic of Korea; 3 School of Traditional Chinese Pharmacy Baoshan College of Traditional Chinese Medicine Baoshan 678000 China

**Keywords:** breast cancer, UPR, RHBDF1, PERK, FoxO3, JNK

## Abstract

Human rhomboid family-1 ( *RHBDF1*) gene is recognized as an oncogene involved in breast cancer development. Previous studies have indicated that RHBDF1 contributes significantly to endoplasmic reticulum (ER) protein homeostasis by stabilizing the binding immunoglobulin protein (BiP) and promoting the unfolded protein response (UPR). Here, we report a relationship between RHBDF1 and the ER stress sensors PERK, IRE1, and ATF6. We show that RHBDF1 deficiency in breast cancer cells results in decreased levels of PERK, pPERK, and peIF2α. These protein levels can be restored in RHBDF1-deficient breast cancer cells by artificial overexpression of RHBDF1 but not IRE1 or ATF6. Additionally, we show that the transcription factor FoxO3 is essential for the RHBDF1-mediated production of PERK. Subsequent analysis reveals that RHBDF1 activates JNK, which causes FoxO3 to translocate into the cell nucleus. These findings demonstrate that RHBDF1 supports the UPR by upregulating the PERK/peIF2α pathway via the JNK/FoxO3 axis and that the functions of RHBDF1 are essential for preserving the homeostasis of ER proteins.

## Introduction

Cancer cells possess an unfolded protein response (UPR) system for survival and growth [ 1- 3] and are resistant to chemotherapy [3]. Cancer cells under endoplasmic reticulum (ER) stress activate the UPR, which is composed of the binding immunoglobulin protein (BiP) and three major branch pathways, including the PRKR-like ER kinase (PERK), inositol-requiring enzyme 1 (IRE1), and activating transcription factor 6 (ATF6) pathways [ 4– 7]. As a primary ER stress sensor, PERK plays a major role in the maintenance of ER protein homeostasis. Under ER stress conditions, PERK becomes dimerized and autophosphorylated, which leads to the phosphorylation of the eukaryotic translation initiation factor 2 subunit (eIF2α) [ 8, 9]. Phosphor-eIF2α temporarily halts global protein synthesis to reduce the ER burden or activate the translation of activating translation factor 4 (ATF4), which in turn activates the apoptotic transcription factor C/EBP homologous protein (CHOP) to induce apoptosis [ 5, 9– 11]. Thus, delineating the molecular mechanism underlying PERK production in cancer cells is important.

Human rhomboid family 1 protein (RHBDF1), also known as inactivated rhomboid 1 (iRhom1), is an ER-resident membrane protein that plays a substantial role in cancer development [ 12 , 13]. Despite the lack of protease activity, the RHBDF1 protein has been found to participate critically in many important biological processes, including stabilizing hypoxia-inducible factor-1α (HIF-1α) in cancer cells under hypoxic conditions [14] and promoting activator protein 1 (AP-1)-activated endothelial-mesenchymal transition (EndMT) by activating the c-Jun N-terminal kinase (JNK) pathway [15]. RHBDF1 expression is diminished in normal breast tissues but highly elevated in tumor tissues, which is strongly correlated with increased disease progression, metastasis, poor prognosis, and poor response to chemotherapy [ 14, 16– 21]. Additionally, RHBDF1 plays a key role in maintaining ER protein homeostasis by stabilizing the BiP protein [22]. Therefore, investigating the involvement of the RHBDF1 protein in the regulation of the functional components of the UPR, namely, the PERK, IRE1, and ATF6 pathways, is worthwhile.

In the present study, we demonstrated that RHBDF1 is able to facilitate PERK expression through the Forkhead box O3 (FoxO3) transcription factor in breast cancer cells. We also showed that RHBDF1 is responsible for FoxO3 translocation into the cell nucleus as a result of JNK pathway activation. Our data are consistent with the view that RHBDF1 functions as an essential part of the UPR and thus plays a critical role in maintaining ER protein homeostasis.

## Materials and Methods

### Reagents and antibodies

Thapsigargin (GC11482; GLPBIO, Montclair, USA) and a JNK inhibitor (SP600125; Beyotime, Shanghai, China) were used in this study. The following antibodies were used: anti-RHBDF1 (ab81342; 1:2000; Abcam, Cambridge, USA), PERK (sc-377400; 1:2000; Santa Cruz Biotechnology, Santa Cruz, USA), anti-phospho-PERK (AF7441; 1:1000; Affinity Biosciences, Cincinnati, USA), anti-phospho-eIF2α (AP0692; 1:1000; ABclonal, Wuhan, China), anti-IRE1 (DF7709; 1:1000; Affinity Biosciences), anti-phospho-IRE1 (AF7150, 1:1000; Affinity Biosciences), anti-ATF6 (DF6009; 1:1000; Affinity Biosciences), anti-FoxO3 (OAGA01231; 1:2000; Aviva Systems Biology, Shanghai, China), anti-phospho-FoxO3 (AP0351; 1:1000; ABclonal), anti-JNK1/2/3 (AF6318; 1:1000; Affinity Biosciences), anti-phospho-JNK1/2/3 (AF3318; 1:1000; Affinity Biosciences), anti-HA (H6533; 1:2000; Sigma-Aldrich, St Louis, USA), anti-FLAG (F7425; 1:2000; Sigma-Aldrich,), anti-β-actin (AC004; 1:5000; ABclonol), anti-histone H3 (4499; 1:2000; CST, Boston, USA), HRP-conjugated goat anti mouse IgG (HS201-01; TransGen Biotech, Beijing, China) and HRP-conjugated goat anti rabbit IgG (HS101-01; TransGen Biotech).

### Cell culture

All cells used in this study were purchased from the American Type Culture Collection (ATCC; Manassas, USA). The stable *RHBDF1*-knockout or *RHBDF1*-knockdown MCF-7, MDA-MB-231, and 4T1 cells used in this study were established in our own lab [15]. The human breast cancer cell line MCF-7 (RRID: CVCL_0031) and human embryonic kidney AD293 cells (RRID: CVCL_9804) were grown in DMEM, and the human breast cancer cell line MDA-MB-231 (RRID: CVCL_0062) and mouse breast cancer cell line 4T1 (RRID: CVCL_0125) were grown in RPMI 1640 medium. All media were supplemented with 10% FBS and penicillin (100 U/mL)/streptomycin (50 μg/mL), and the cells were grown at 37°C in 5% CO _2_.

### Cell transfection

Full-length human RHBDF1 tagged with C-terminal HA and full-length human FoxO3 tagged with C-terminal FLAG were cloned and inserted into pLVX-EF1α-IRES-Puro (LM-2015; LMAI Bio, Shanghai, China). The plasmids were transfected into MCF-7 cells using Lipofectamine 2000 (Thermo Fisher Scientific, Waltham,, USA) with reduced serum medium Opti-MEM (Life Technologies, Carlsbad, USA) following the manufacturer’s instructions.

### Western blot analysis

MCF-7, MDA-MB-231, or 4T1 cells were cultured in cell culture plates at 37°C in 5% CO _2_. The cells were lysed in RIPA buffer supplemented with 1% protease inhibitors (P8215; Sigma-Aldrich). For the nuclear-cytoplasmic protein separation assay, we used Nuclear and Cytoplasmic Protein Extraction Kit from Beyotime (#P0028). After separation via SDS-PAGE and transfer to PVDF membranes (IPFL00010; Millipore, Billerica, USA), the membranes were blocked in PBST with 5% nonfat dry milk powder for 1 h at room temperature, followed by incubation overnight at 4°C with primary antibodies against the target proteins. On the second day, the membrane was washed 3 times with PBST for 10 min and then further incubated with the appropriate horseradish peroxidase (HRP)-conjugated secondary antibody for 1 h at room temperature. The membrane was washed as described above, and protein expression was detected using an enhanced chemiluminescence (ECL) solution.

### Quantitative RT-PCR

cDNA was obtained from the indicated cells via reverse transcription of total RNA extracted with Trizol (Invitrogen, Carlsbad, USA) from indicated cells using SuperScript III First-Strand Synthesis SuperMix (11752-50; Invitrogen). One microliter of the reverse transcription product and 250 nM of each primer were added to a 20-μL volume reaction mixture, which was denatured at 95°C for 15 s, annealed, and extended at 60°C for 30 s. The comparative 2 ^–ΔΔCt^ method was used to calculate the relative quantity of each sample, which was normalized to that of *GAPDH*. The primers used for the indicated gene products are listed in Table 1 .

**Table:** **Table 1** The sequences of primers used for real-time PCR

Name	Primer sequence (5′→3′)
Human *PERK* forward	GGAAACGAGAGCCGGATTTATT
Human *PERK* reverse	ACTATGTCCATTATGGCAGCTTC
Human *IRE1* forward	AGAGAAGCAGCAGACTTTGTC
Human *IRE1* reverse	GTTTTGGTGTCGTACATGGTGA
Human *ATF6* forward	TCCTCGGTCAGTGGACTCTTA
Human *ATF6* reverse	CTTGGGCTGAATTGAAGGTTTTG
Human *GAPDH* forward	AAGCCTGCCGGTGACTAAC
Human *GAPDH* reverse	GTTAAAAGCAGCCCTGGTGAC

### Immunofluorescence staining

MCF-7 MT/R1KO cells were attached to a glass slide in a 24-well cell culture dish and cultured at 37°C and 5% CO _2_ for 6–12 h. The slide was washed three times for 5 min with PBS, fixed with 4% paraformaldehyde for 15 min, and then permeabilized with 0.3% Triton X-100 for 20 min. Then, it was blocked with 5% bovine serum albumin for 1 h and incubated with primary antibodies overnight at 4°C. After the samples were washed with PBST 3 times for 5 min, they were incubated with a secondary antibody for 1 h at room temperature and then stained with DAPI (33342; Invitrogen). The samples were sealed with antifading mounting medium and imaged with an LSM 800 instrument (Carl Zeiss, Oberkochen, Germany).

### Statistical analysis

GraphPad Prism 8 was used for data analysis. All data were obtained from at least three independent experiments and are presented as the mean ± SD. Statistical tests were performed via the unpaired two-tailed Student’s *t* test. The survival curves and their statistical tests were calculated via the Kaplan-Meier method and the log-rank test. *P* values less than 0.05 were considered to be statistically significant.

## Results

### RHBDF1 deficiency results in downregulation of the PERK/peIF2α pathway in breast cancer cells

**Figure 1:**
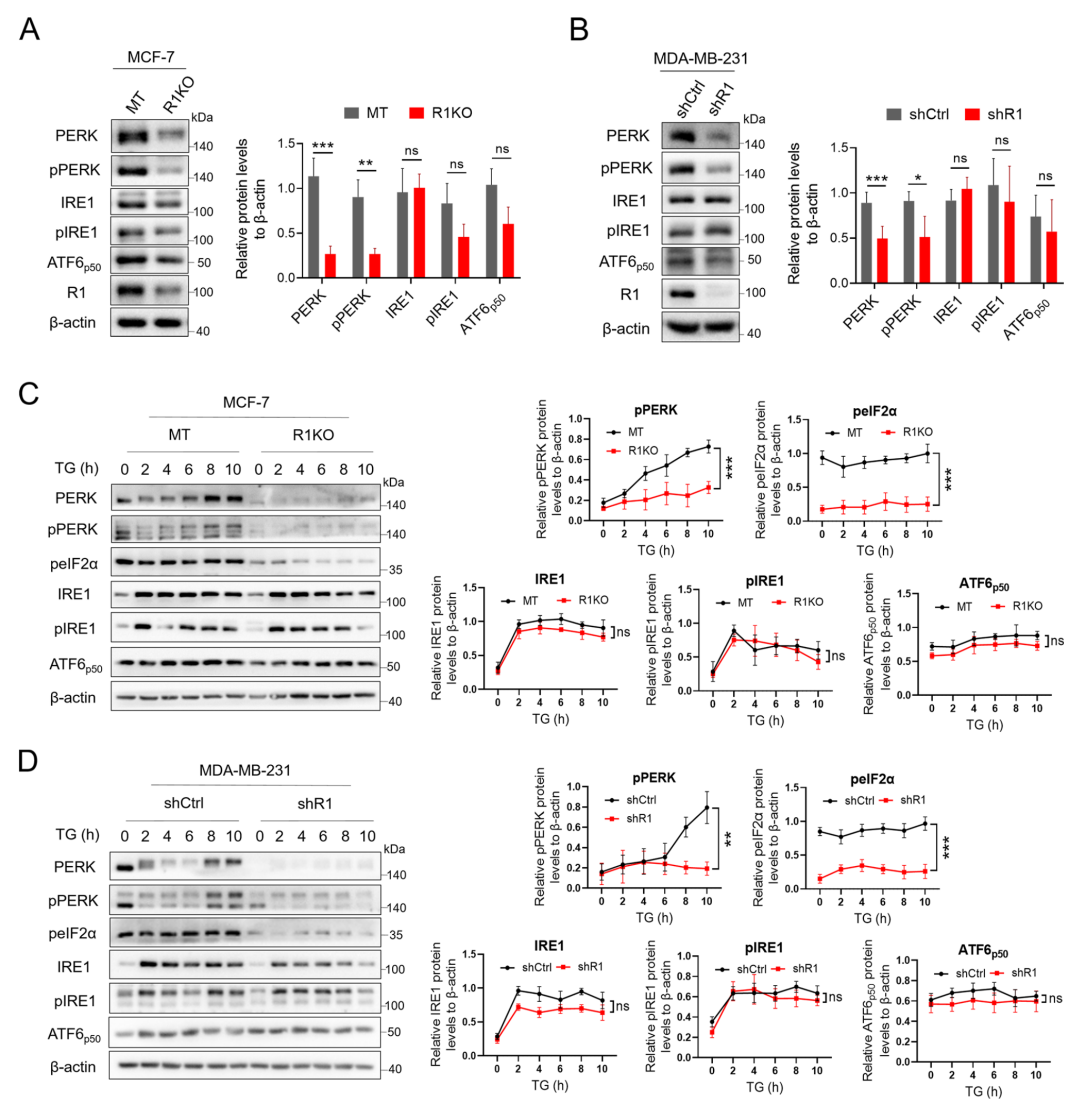
Figure 1 Loss of RHBDF1 downregulates the PERK/peIF2α pathway in breast cancer cells (A,B) Western blot analysis of ER stress sensors in MCF-7 MT/R1KO (A) and MDA-MB-231 shCtrl/shR1 (B) breast cancer cells. (C,D) Western blot analysis of ER stress sensors in MCF-7 MT/R1KO (C) and MDA-MB-231 shCtrl/shR1 (D) breast cancer cells treated with thapsigargin (TG) (2 μM). Representative images (left) and data summaries (right) from three experiments are shown. Data are presented as the mean ± SD, n = 3. *P < 0.05, **P < 0.01, ***P < 0.001.

To investigate the role of RHBDF1 in the UPR, we determined the protein levels of ER stress sensors in *RHBDF1*-knockout MCF-7 and 4T1 breast cancer cells. Western blot analysis revealed that the protein levels of PERK, phosphorylated PERK, and phosphorylated eIF2α were markedly lower in RHBDF1-deficient cells (R1KO) than in mock-transfected (MT) control cells; however, the protein levels of IRE1 and ATF6 remained unchanged ( Figure 1A and Supplementary Figure S1). Similar results were obtained when the experiments were repeated in MDA-MB-231 cells, in which the *RHBDF1* gene was silenced with shRNA against RHBDF1 (shR1, F: 5′-CCGGCAGTGACAGCACCCAGAAATGCTCGAGCATTTCTGGGTGCTGTCACTGTTTTTTG-3′; R: 5′-AATTCAAAAACCGGCAGTGACAGCACCCAGAAATGCTCGAGCATTTCTGGGTGCTGTCACTG-3′) ( Figure 1B). Additionally, when cancer cells were cultured in the presence of the ER stress inducer thapsigargin (TG), the phospho-PERK protein level increased gradually in MT- or shCtrl-treated control cells but not in RHBDF1-deficient cells ( Figure 1C,D). Moreover, we found that downregulation of phospho-eIF2α was accompanied by the downregulation of phospho-PERK but not by IRE1 or ATF6 ( Figure 1C,D). These findings indicated that the RHBDF1 protein is critically involved in the modulation of the PERK/peIF2α pathway in the UPR.

### Artificially overexpressing RHBDF1 can restore PERK protein levels in RHBDF1-deficient breast cancer cells

**Figure 2:**
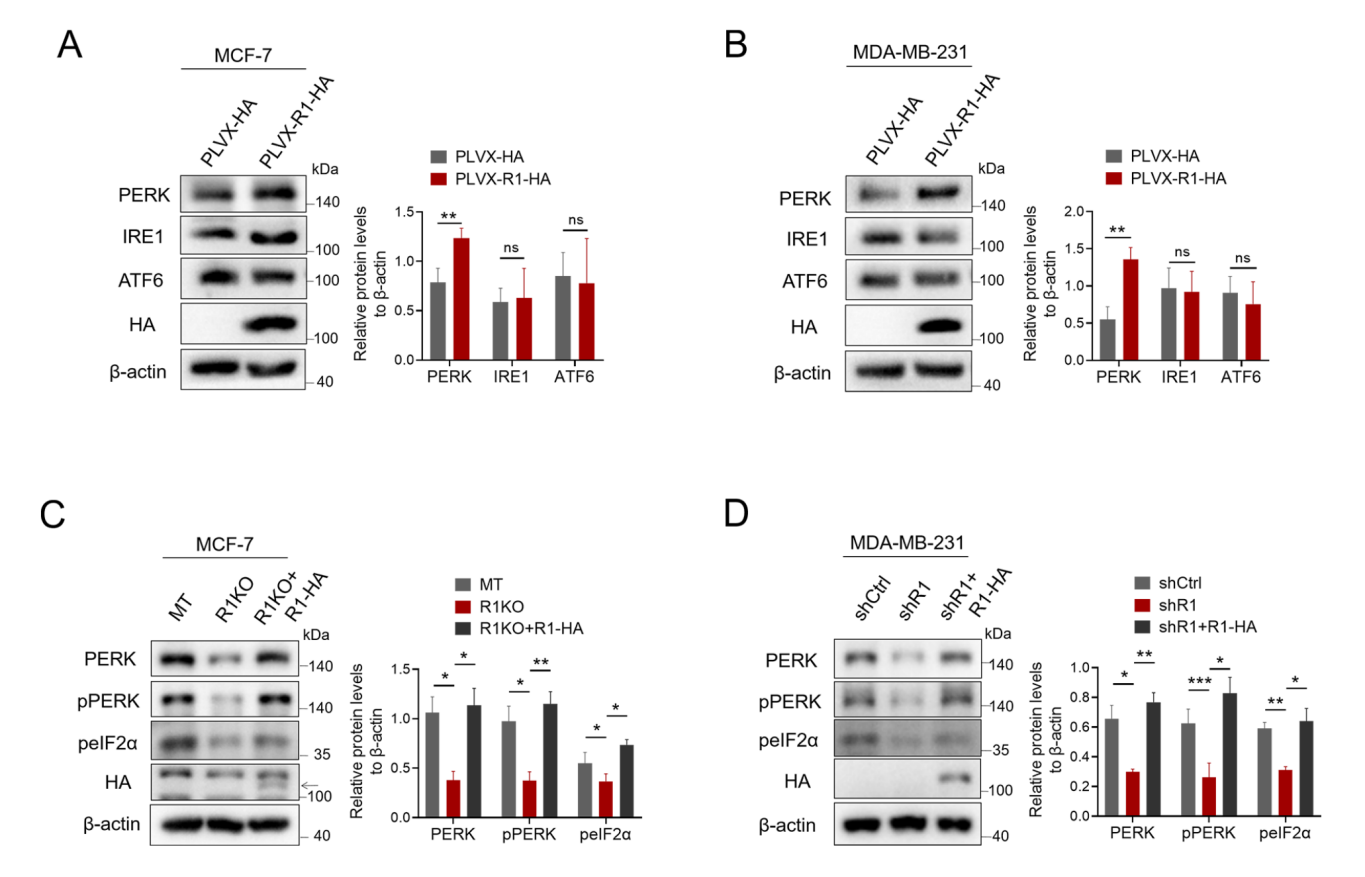
Figure 2 Overexpression of RHBDF1 reverses the decrease in the levels of PERK and its downstream proteins in breast cancer cells lacking RHBDF1 (A,B) Western blot analysis of ER stress sensors in MCF-7 (A) and MDA-MB-231 (B) breast cancer cells overexpressing RHBDF1. (C,D) Western blot analysis of PERK, pPERK, and peIF2α in RHBDF1-knockout MCF-7 (C) and shR1-treated MDA-MB-231 (D) breast cancer cells overexpressing RHBDF1. Representative images (left) and data summaries (right) from three experiments are shown. Data are presented as the mean ± SD, n = 3. *P < 0.05, ** P < 0.01, ***P < 0.001.

We created a plasmid of RHBDF1 tagged with C-terminal HA, transfected it temporarily into MCF-7 breast cancer cells, and measured the protein levels of ER stress sensors in the cells to examine the impact of overexpressing RHBDF1. By using western blot analysis, it was possible to observe that MCF-7 cells that were artificially overexpressing RHBDF1 had much higher amounts of PERK protein than control cells that were overexpressing the empty vector; however, there was no significant increase in IRE1 or ATF6 protein ( Figure 2A). Similar outcomes were also observed in MDA-MB-231 and 4T1 cells that were transiently overexpressing either the empty vector or RHBDF1 tagged with C-terminal HA ( Figure 2B and Supplementary Figure S2). Next, we assessed the protein levels of PERK and its downstream phospho-PERK and phospho-eIF2α in *RHBDF1*-knockout MCF-7 and 4T1 breast cancer cells overexpressing C-terminal HA-tagged RHBDF1. We discovered that in *RHBDF1*-knockout MCF-7 and 4T1 cells, artificial overexpression of RHBDF1 restored the reduced protein levels of PERK as well as its downstream phospho-PERK and phospho-eIF2α ( Figure 2C and Supplementary Figure S3). These recoveries were also observed in MDA-MB-231 cells treated with shR1 when RHBDF1 was temporarily overexpressed ( Figure 2D). These findings showed that the RHBDF1 protein plays a crucial role in regulating the PERK/peIF2α pathway in the UPR.

### RHBDF1 promotes PERK transcription through FoxO3

**Figure 3:**
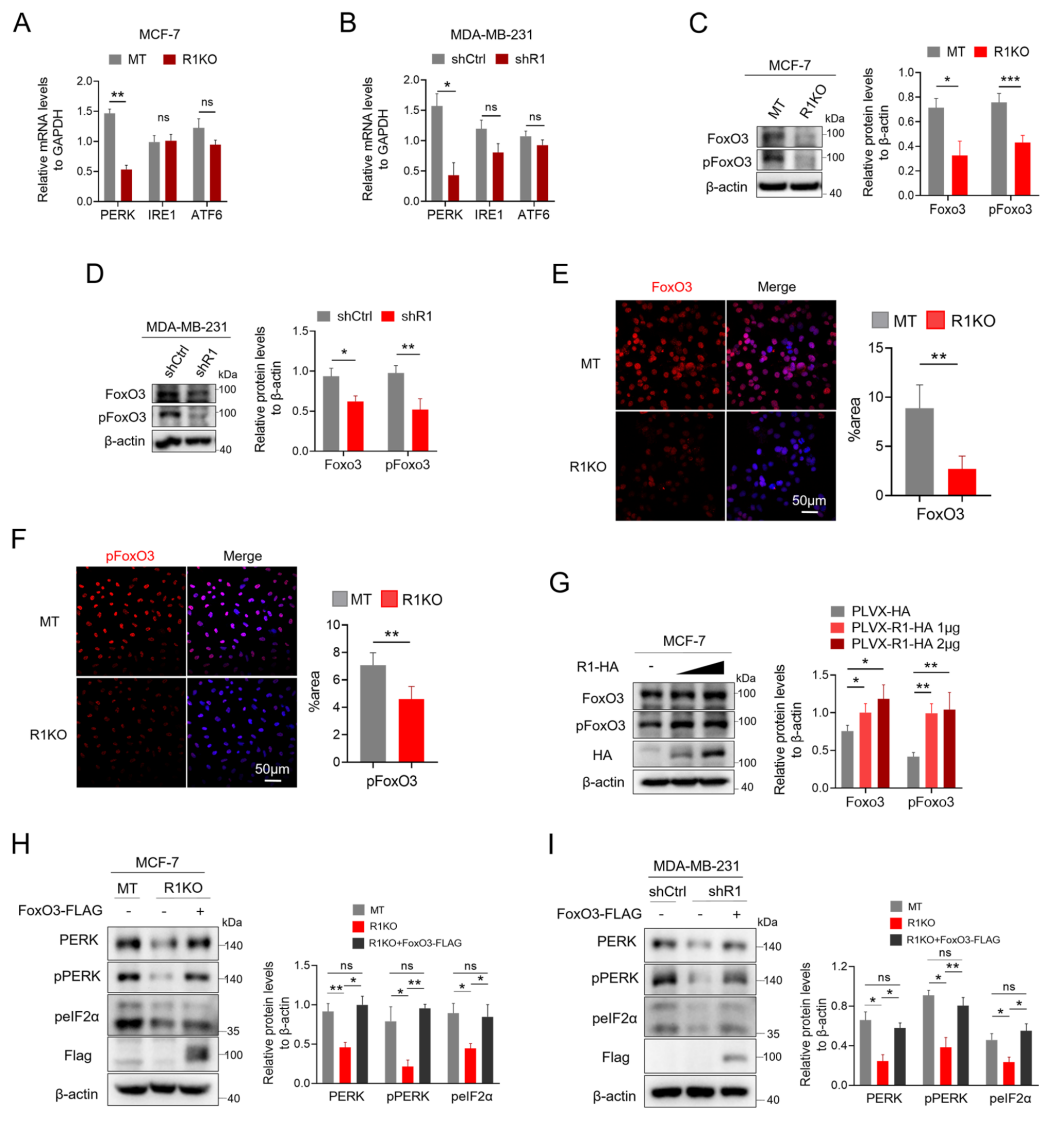
Figure 3 RHBDF1 regulates PERK expression through FoxO3 (A,B) RT-qPCR analysis of ER stress sensors in MCF-7 MT/R1KO cells (A) and MDA-MB-231 shCtrl/shR1 cells (B). (C,D) Western blot analysis of FoxO3 and pFoxO3 in MCF-7 MT/R1KO cells (C) and MDA-MB-231 shCtrl/shR1 cells (D). (E,F) Immunofluorescence staining of MCF-7 MT/R1KO cells with anti-FoxO3 (E) or anti-phospho-FoxO3 (F) antibodies. (G) Western blot analysis of FoxO3 and pFoxO3 in MCF-7 cells overexpressing RHBDF1-HA. (H,I) Western blot analysis of PERK and its downstream proteins in RHBDF1-knockout MCF-7 cells (H) and RHBDF1-knockdown MDA-MB-231 cells (I) overexpressing FoxO3-FLAG. Representative images (left) and data summaries (right) from three experiments are shown. Data are presented as the mean ± SD, n = 3. *P < 0.05, ** P < 0.01.

Using RT-qPCR analysis, we assessed the mRNA levels of *PERK* in MCF-7 MT/R1KO and MDA-MB-231 shCtrl/shR1 breast cancer cells to explore the mechanism by which RHBDF1 stimulates the synthesis of the PERK protein. Compared with those in control cells, *PERK* mRNA levels but not *IRE1* or *ATF6* mRNA levels were considerably lower in *RHBDF1*-knockout MCF-7 and *RHBDF1*-silenced MDA-MB-231 breast cancer cells ( Figure 3A,B). The same results were also obtained in the 4T1 MT/R1KO cells ( Supplementary Figure S4). These findings suggested that RHBDF1 may be connected to PERK protein transcription. FoxO3 is reportedly an upstream PERK transcription factor [23]. We also examined the protein levels of FoxO3 and phospho-FoxO3 in breast cancer cells lacking RHBDF1. Western blot analysis revealed that *RHBDF1*-knockout MCF-7 cells had considerably lower levels of FoxO3 and phospho-FoxO3 than control cells ( Figure 3C). Similar results were also obtained in MDA-MB-231 shCtrl/shR1 and 4T1 MT/R1KO breast cancer cells by western blot analysis ( Figure 3D and Supplementary Figure S5).

By using immunofluorescence staining, we also demonstrated a decrease in FoxO3 and phospho-FoxO3 in *RHBDF1*-knockout MCF-7 and *RHBDF1*-silenced MDA-MB-231 breast cancer cells ( Figure 3E,F). Additionally, we measured the amounts of FoxO3 and phospho-FoxO3 proteins in MCF-7 cells after transient transfection with the C-terminal HA-tagged RHBDF1 plasmid. By comparing RHBDF1-overexpressing MCF-7 cells to empty vector-overexpressing control cells, we discovered that the protein levels of FoxO3 and phospho-FoxO3 were dramatically increased in a dose-dependent manner ( Figure 3G). These results suggested that the RHBDF1 protein is involved in the regulation of FoxO3 transcriptional activity. We constructed a C-terminal Flag-tagged FoxO3 plasmid and transiently transfected it into *RHBDF1*-knockout MCF-7 cells to determine the protein level of PERK in the cells. Western blot analysis demonstrated that artificial overexpression of FoxO3 restored the decrease in PERK protein level in *RHBDF1*-knockout MCF-7 cells ( Figure 3H). Furthermore, the restoration of PERK protein level also resulted in the restoration of the downstream proteins phospho-PERK and phospho-eIF2α ( Figure 3H). Moreover, the same results were obtained from the western blot analysis of MDA-MB-231 and 4T1 cells overexpressing FoxO3 ( Figure 3I and Supplementary Figure S6). These findings indicated that RHBDF1 stimulates the transcription factor FoxO3, which in turn supports the PERK/peIF2α pathway in breast cancer cells by promoting PERK transcription.

### RHBDF1 is responsible for FoxO3 translocation into the cell nucleus by activating JNK

**Figure 4:**
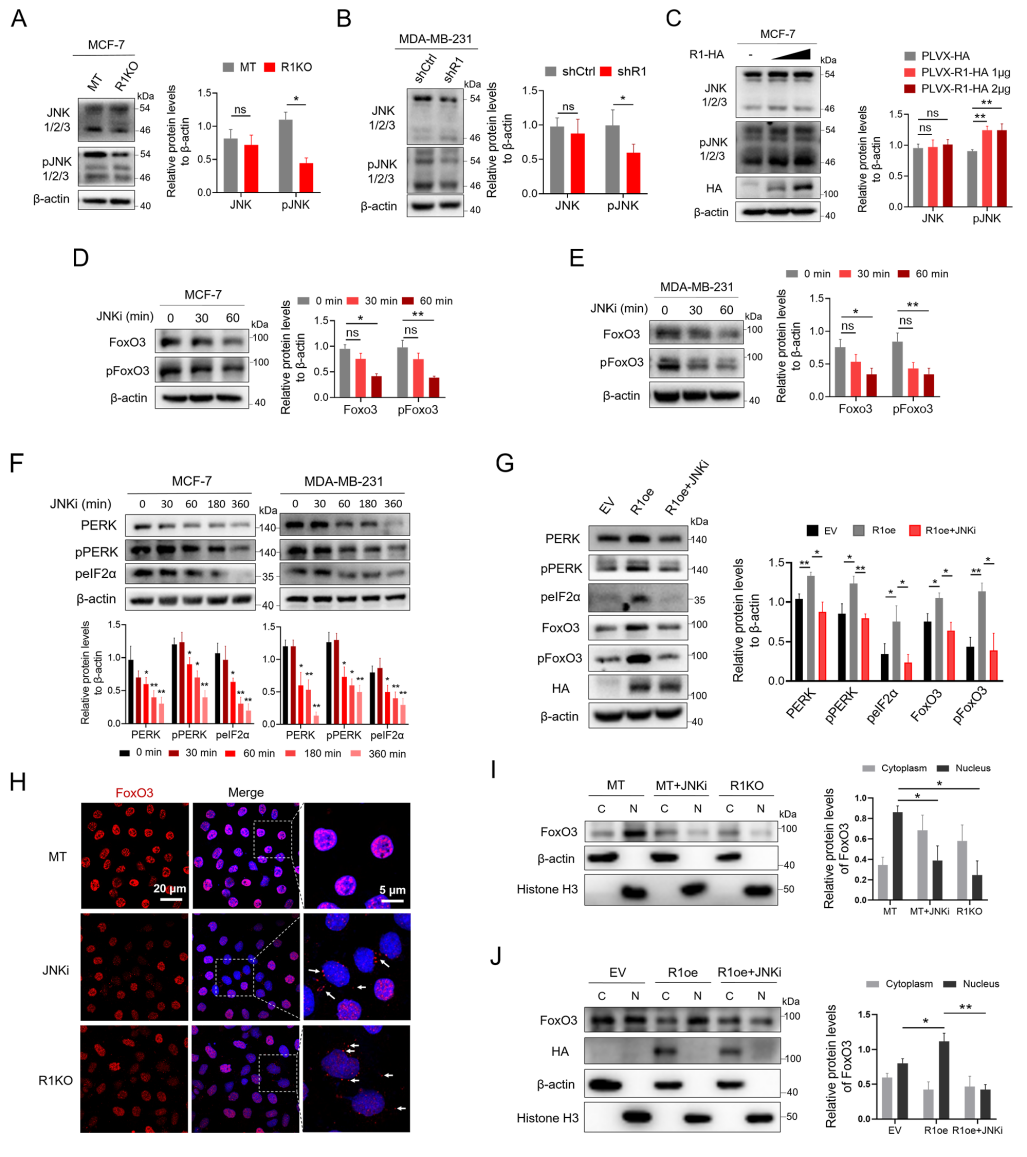
Figure 4 RHBDF1 promotes FoxO3 translocation into the cell nucleus by activating JNK in breast cancer cells (A,B) Western blot analysis of JNK and pJNK in MCF-7 (MT and R1KO) cells (A) and MDA-MB-231 (shCtrl and shR1) cells (B). (C) Western blot analysis of JNK and pJNK in RHBDF1-HA-overexpressing MCF-7 cells. Representative images (left) and data summaries (right) from three experiments are shown. (D,E) Western blot analysis of FoxO3 and pFoxO3 in MCF-7 cells (D) and MDA-MB-231 cells (E) treated with JNKi (10 μM). Representative images (left) and data summaries (right) from three experiments are shown. (F) Western blot analysis of the PERK/peIF2α pathway in breast cancer cells treated with JNKi (10 μM). Representative images (up) and data summaries (down) from three experiments are shown. (G) Western blot analysis of the PERK/peIF2α and FoxO3/pFoxO3 pathways in RHBDF1-HA-overexpressing MCF-7 cells with or without JNKi treatment (10 μM, 2 h). Representative images (left) and data summaries (right) from three experiments are shown. (H) Immunofluorescence staining with anti-FoxO3 antibodies for determining FoxO3 sublocalization in JNKi-treated MCF-7 cells and RHBDF1 knockout MCF-7 cells. The control MCF-7 cells (MT) were treated with JNKi (10 μM) for 2 h. (I) Western blot analysis of FoxO3 in the cytoplasm and nucleus of JNKi (10 μM, 2 h)-treated MCF-7 cells and RHBDF1-knockout MCF-7 cells. Representative images (left) and data summaries (right) from three experiments are shown. (J) Western blot analysis of FoxO3 in the cytoplasm and nucleus of RHBDF1-overexpressing MCF-7 cells with or without JNKi treatment (10 μM, 2 h). Representative images (left) and data summaries (right) from three experiments are shown. Data are presented as the mean ± SD, n = 3. * P < 0.05, **P < 0.01.

JNK phosphorylates FoxO3 and promotes its translocation into the cell nucleus [ 24- 26], and RHBDF1 activates JNK in breast cancer cells [15]. We hypothesized that RHBDF1 may activate JNK, which would then aid in the translocation of FoxO3 into the cell nucleus. We initially determined the connection between RHBDF1 and JNK in human breast cancer cells to support this hypothesis. Phospho-JNK, but not JNK, was dramatically lower in *RHBDF1*-knockout MCF-7 or *RHBDF1*-knockdown MDA-MB-231 breast cancer cells than in control cells, according to western blot analysis ( Figure 4A,B). After RHBDF1 was temporarily overexpressed in MCF-7 cells, we used western blot analysis to measure the protein levels of JNK and phospho-JNK in the cells. The results demonstrated that phospho-JNK protein level in MCF-7 cells was significantly elevated by artificial overexpression of RHBDF1 ( Figure 4C). These results supported the finding that RHBDF1 controls JNK activation in breast cancer cells [15]. Moreover, we treated MCF-7 and MDA-MB-231 cells with a JNK inhibitor (JNKi) and measured the protein levels of FoxO3 and phospho-FoxO3 in the cells. Through the use of western blot analysis, we found that, compared with those in control cells, the protein levels of FoxO3 and phospho-FoxO3 in MCF-7 or MDA-MB-231 breast cancer cells were dramatically lower in the JNKi-treated cells ( Figure 4D,E). We treated breast cancer cells with a JNKi for various durations and measured the levels of PERK and its downstream proteins to examine the impact of JNK on the PERK/peIF2α pathway. We discovered that JNKi treatment greatly reduced the levels of PERK and its downstream phospho-PERK and phospho-eIF2α proteins in breast cancer cells in a time-dependent manner ( Figure 4F). After the JNKi was administered to RHBDF1-overexpressing MCF-7 and 4T1 cells, we measured the protein levels of FoxO3 and PERK via western blot analysis. We discovered that JNKi treatment reversed the increase in PERK, phospho-PERK, phospho-eIF2α, FoxO3, and phospho-FoxO3 in MCF-7 and 4T1 cells overexpressing RHBDF1 ( Figure 4G and Supplementary Figure S7). These findings showed that RHBDF1-modulated PERK expression depends on the JNK/FoxO3 pathway. We investigated the involvement of RHBDF1 in FoxO3 translocation in breast cancer cells to better understand the mechanism by which RHBDF1 controls FoxO3 transcriptional activity. Through the use of immunofluorescence staining, we discovered that both JNK inhibition and RHBDF1 deletion significantly reduced the amount of FoxO3 protein in the cell nucleus compared with that in control cells ( Figure 4H). Additionally, FoxO3 protein levels in the nucleus of *RHBDF1*-knockout MCF-7 and 4T1 cells were lower than those in the nucleus of control cells, as demonstrated by western blot analysis ( Figure 4I and Supplementary Figure S8). Furthermore, FoxO3 protein levels in the nucleus of MCF-7 and 4T1 cells were elevated by artificial overexpression of RHBDF1, and these elevated levels were reversed in RHBDF1-overexpressing MCF-7 cells by treatment with a JNKi ( Figure 4J and Supplementary Figure S9). These findings demonstrated that RHBDF1 activates JNK, which causes FoxO3 to translocate into the cell nucleus.

## Discussion

The ER is an important organelle with an extensive network structure and responds strongly to various abnormal cellular microenvironmental stresses [ 27, 28]. In particular, the ability of the ER to maintain protein homeostasis, which is achieved via the UPR, is critical for cancer cell survival and development [ 8, 29, 30]. RHBDF1, an oncogene protein, is critically involved in various biological processes in the tumor microenvironment [ 21, 31]. Many studies have shown that RHBDF1 plays a key role in cancer progression, chemotherapy resistance, and poor prognosis [ 14, 18, 19, 32, 33,37], but the underlying molecular mechanism remains unclear. Our previous study demonstrated that RHBDF1 is essential for maintaining ER protein homeostasis by stabilizing BiP [22]. In addition to the chaperone protein BiP, three primary branches, the PERK, IRE1, and ATF6 pathways, are involved in the UPR [ 2, 34]. These pathways are crucial for the UPR to transiently halt global translation, synthesize new chaperone proteins, and induce autophagy or apoptosis [ 35, 36], but the relationships between RHBDF1 and the three UPR branch pathways have not been studied. As key regulators of the UPR, the roles of the ER stress sensors PERK, IRE1, and ATF6 have been well studied, but the specific molecular mechanisms related to their protein production have not yet been explored.

**Figure 5:**
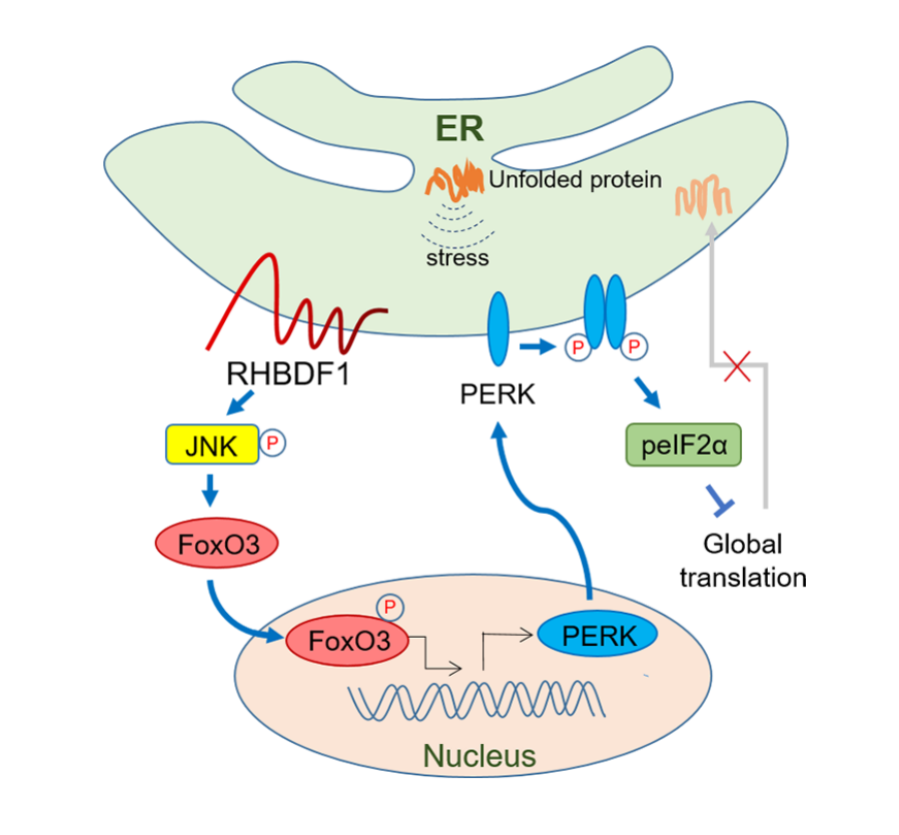
Figure 5 Schematic diagram of how RHBDF1 mediates PERK expression through the JNK/FoxO3 axis RHBDF1 activates JNK, causing FoxO3 to be activated and translocated into the cell nucleus, resulting in the promotion of PERK expression and thereby controlling global translation by the upregulation of the PERK/peIF2α pathway.

In this study, as shown in Figure 5, we found that RHBDF1 supports the UPR by promoting FoxO3-activated PERK expression. This is at least achieved by RHBDF1 promoting FoxO3 translocation into the cell nucleus through the activation of JNK. The PERK/peIF2α pathway is a branch of the UPR that plays an integral role in ER protein homeostasis by regulating global translation. According to our results, PERK expression depends on the RHBDF1-mediated JNK/FoxO3 axis. RHBDF1 deficiency leads to decreased JNK-mediated FoxO3 translocation into the cell nucleus, resulting in a reduction in PERK and its downstream proteins pPERK and peIF2α in breast cancer cells. These findings suggested that RHBDF1 deficiency can result in uncontrolled global translation to induce ER stress. These results are also consistent with our previous findings that *RHBDF1* knockdown or knockout results in marked unfolded protein aggregation and ER stress [22]. Our findings shed light on the molecular mechanism underlying the expression of PERK and indicate that RHBDF1 plays a substantial role in supporting the UPR not only by stabilizing BiP [22] but also by regulating the PERK/peIF2α pathway, which may be one of the main mechanisms by which RHBDF1 promotes breast cancer development and chemotherapy resistance.

## Supplementary Data

Supplementary Data is available at *Acta Biochimica et Biophysica Sinica* online.
